# Phylogenomic analysis for *Campylobacter fetus* ocurring in Argentina

**DOI:** 10.14202/vetworld.2021.1165-1179

**Published:** 2021-05-13

**Authors:** Pablo Daniel Farace, José Matías Irazoqui, Claudia Graciela Morsella, Juan Agustín García, María Alejandra Méndez, Fernando Alberto Paolicchi, Ariel Fernando Amadio, Andrea Karina Gioffré

**Affiliations:** 1 Instituto de Agrobiotecnología y Biología Molecular, Instituto Nacional de Tecnología Agropecuaria-Consejo Nacional de Investigaciones Científicas y Técnicas (IABIMO INTA-CONICET), Hurlingham, Buenos Aires, Argentina; 2 Consejo Nacional de Investigaciones Científicas y Tecnológicas (CONICET), Estación Experimental Agropecuaria-INTA, Rafaela, Santa Fe, Argentina; 3 Laboratorio de Bacteriología-Grupo de Sanidad Animal. Unidad Integrada INTA-Universidad Nacional de Mar del Plata, Balcarce, Buenos Aires, Argentina

**Keywords:** *Campylobacter fetus*, multi-locus sequence typing, pangenome, phylogenomics, venereal disease

## Abstract

**Background and Aim:**

*Campylobacter fetus* is one of the most important pathogens that severely affects livestock industry worldwide. *C. fetus* mediated bovine genital campylobacteriosis infection in cattle has been associated with significant economic losses in livestock production in the Pampas region, the most productive area of Argentina. The present study aimed to establish the genomic relationships between *C. fetus* strains, isolated from the Pampas region, at local and global levels. The study also explored the utility of multi-locus sequence typing (MLST) as a typing technique for *C. fetus*.

**Materials and Methods:**

For pangenome and phylogenetic analysis, whole genome sequences for 34 *C. fetus* strains, isolated from cattle in Argentina were downloaded from GenBank. A local maximum likelihood (ML) tree was constructed and linked to a Microreact project. *In silic*o analysis based on MLST was used to obtain information regarding sequence type (ST) for each strain. For global phylogenetic analysis, a core genome ML-tree was constructed using genomic dataset for 265 *C. fetus* strains, isolated from various sources obtained from 20 countries.

**Results:**

The local core genome phylogenetic tree analysis described the presence of two major clusters (A and B) and one minor cluster (C). The occurrence of 82% of the strains in these three clusters suggested a clonal population structure for *C. fetus*. The MLST analysis for the local strains revealed that 31 strains were ST4 type and one strain was ST5 type. In addition, a new variant was identified that was assigned a novel ST, ST70. In the present case, ST4 was homogenously distributed across all the regions and clusters. The global analysis showed that most of the local strains clustered in the phylogenetic groups that comprised exclusively of the strains isolated from Argentina. Interestingly, three strains showed a close genetic relationship with bovine strains obtained from Uruguay and Brazil. The ST5 strain grouped in a distant cluster, with strains obtained from different sources from various geographic locations worldwide. Two local strains clustered in a phylogenetic group comprising intercontinental *Campylobacter fetus venerealis* strains.

**Conclusion:**

The results of the study suggested active movement of animals, probably due to economic trade between different regions of the country as well as with neighboring countries. MLST results were partially concordant with phylogenetic analysis. Thus, this method did not qualify as a reliable subtyping method to assess *C. fetus* diversity in Argentina. The present study provided a basic platform to conduct future research on *C. fetu*s, both at local and international levels.

## Introduction

Argentina is the sixth-largest exporter of meat in the world (http://www.worldstopexports.com/top-beef-exporting-countries/). Since ancient times, livestock farming has been an important traditional activity in Argentina and it is majorly practiced in the fertile pastures of the Pampas (Buenos Aires province and its surrounding area, Argentina). Livestock production is one of the most important contributors of the economy in Argentina. Recent times have witnessed an increase in local and international demands for animal protein, demanding a substantial improvement in livestock productivity. Bovine reproductive infections are one of the major challenges faced by livestock industry. Such infections account for significant financial losses every year. *Campylobacter fetus* infection has been identified as the main cause of bovine abortions in the Pampas [1,2]. Among the various subspecies of *C. fetus*, *Campylobacter fetus fetus* (Cff) and *Campylobacter fetus venerealis* (Cfv) are two most important subspecies that are associated with poor reproductive health in cattle. In particular, these two subspecies have been reported to significantly affect herd reproductive parameters. Cfv is linked to bovine genital campylobacteriosis, a venereal disease that is primarily associated with infertility. *C. fetus* biovar intermedius, a Cfv variant that shares intermediate biochemical traits with Cfv and Cff, has been found to be frequently associated with late abortions in cattle. Several previous studies have reported the presence of Cfvi in cattle in Argentina. The diagnosis of the disease can be done by evaluating genital secretions of cows and bulls. In addition to this, aborted fetus and placental tissues can also be used for the diagnosis. Despite the advances in the molecular methods used for the identification and differentiation of subspecies, inconsistencies in the outcome poses a great challenge [3,4]. In South America, direct immunofluorescence-based screening assay is the method of choice for the identification of the pathogen and elimination of the infected animals from endemic herds [5]. This method involves direct detection of *C. fetus* with the aid of hyperimmune sera that are raised using total antigens of the bacterium. However, the inability of this method to differentiate between various subspecies limits its application. A third subspecies, *C. fetus* subsp. *testudinum*, has been identified in reptiles and humans. In humans, it has been isolated from various sources including feces, blood, pleural, and bile [6]. There is no evidence for the presence of this subspecies in Argentina; however, this could be attributed to limited study of the samples. This subspecies has not been identified in ­cattle so far. Despite the absence of official epidemiological data, *C. fetus* has been found to be associated with bacteremia in immunocompromised patients in Argentina [7,8]. However, no information is currently available regarding the subspecies of *C. fetus* responsible for human infections in Argentina, probably due to the lack of reliable and sophisticated tools. This highlights the need for the immediate development of accurate methods for the identification of *C. fetus* subspecies.

Since 1990, the Laboratory of Bacteriology of EEA-INTA Balcarce, Argentina has isolated *C. fetus* from various veterinary samples. The organization has provided a differential diagnosis for the identification of this pathogen. They have collected 250 strains of the pathogen in the past 30 years. According to the data provided by the Laboratory of Bacteriology, Cff and Cfv are the most prevalent subspecies that are responsible for bovine abortion. In a recent study by our group, whole-genome sequencing of *C. fetus* was performed utilizing the services provided by the Genomic Unit of INTA. The study aimed to characterize *C. fetus* strains and thus contribute to the bulk of genomic sequences of *C*. *fetus* strains found in Argentina, that are currently available in public databases.

In the present study, phylogenomic analysis was performed to gain better insights into the global and local patterns for the spread of this pathogen, with a view to improve its surveillance. The present study aimed to extend the currently available knowledge regarding the different strains of *C. fetus* found in Argentina and promotes collaborative research between various groups from animal as well as human health sectors. The results of the study will provide a better and wholistic understanding regarding the local and regional epidemiological scenario involving this bacterium.

## Materials and Methods

### Ethical approval

This study doesn´t need ethical approval. This is a genomic dataset-based study.

### Study period and location

The study was conducted from October 2019 to August 2020 at the research units of the National Agricultural Technology Institute (INTA), Argentina.

### Whole genome sequences and pangenome analysis

For phylogenomic analysis, 34 freely available complete genome sequences for bovine *C. fetus* strains, isolated from the Pampas region, were downloaded from GenBank (last access to the database: August 2020). The present study included strains from four provinces of the Pampas region, namely, Buenos Aires (n=27), Santa Fe (n=2), La Pampa (n=2), and Córdoba (n=2) (Supplementary Table-1). The origin of one strain remained unknown. These strains were isolated over a period of 26 years (1989-2015) from various sources, including prepuce, placenta, vaginal mucus, and fetus. For analysis, genomic sequences were assembled using SPAdes 3.11.1 [9]. Data filtering for contigs <200 bp and the ones with low coverage (<10) resulted in 96 contigs per genome on average.

For the global phylogenetic study, all publicly available *C. fetus* whole-genome sequences were used (n=265) (last access to GenBank: August 2020). These sequences represented *C. fetus* strains obtained from different hosts and from 20 countries, including Australia (n=4), Belgium (n=1), Brazil (n=2), Canada (n=19), China (n=12), Czech Republic (n=1), France (n=39), Germany (n=19), Ireland (n=1), Italy (n=2), the Netherlands (n=12), New Zealand (n=1), South Africa (n=4), Spain (n=31), Turkey (n=1), Taiwan (n=20), the United Kingdom (n=36), the United States of America (n=19), Uruguay (n=5), Argentina (n=34) and two strains were of unknown origin (associated data in Supplementary Table-2) [10]. For analysis, PROKKA was used as annotation tool for the assembled genomes [11]. GFF3 files were used as input and the pangenome were obtained using the pipeline Roary [12] that allows rapid large-scale prokaryote pangenome analysis (threshold of sequence identity ≥90%).

### Phylogenomic analysis

MAFFT was used for core genome multiple sequence alignment. A maximum likelihood (ML) phylogenetic tree (ML tree) was generated using IQ-Tree 1.6.12 [13], which was further visualized with the aid of iTol v5 [14]. The node support was evaluated with 1000 bootstrap pseudoreplications. Subsequently, a heatmap was generated in R using the ‘Matrix’ package software (https://CRAN.R-project.org/package=Matrix) for pairwise comparison of all the strains included in the study.

### Multi-locus sequence typing (MLST)

*In silico* MLST (https://github.com/tseemann/mlst) was performed to obtain the sequence type (ST) of each strain obtained from Argentina. Each genome was scanned against the traditional PubMLST typing scheme (Campylobacter non jejuni/coli PubMLST database). In the cases where inconclusive results were obtained, pipeline MLST 2.0 (https://cge.cbs.dtu.dk/services/MLST/) was also employed for analysis. The novel allelic variants and their respective ST were deposited in the PubMLST database (https//:www.pubMLST.org).

### Visualization of phylogenetic tree linked to metadata using Microreact

Microreact software [15] was used to visualize the local phylogenetic tree in a spatial and temporal context. A public project was created through the Microreact homepage. Metadata were collected from historical data collection of INTA (host, source, year of isolation, biochemical, and molecular traits of the strains) (Supplementary Table-1). Geno‐ and phenotyping of local strains were performed according to the previously published protocols [10,16]. Geodata were obtained with the aid of Google Maps (https//:maps.google.com).

## Results

### Pangenome analysis of local *C. fetus* strains

**Figure-1:**
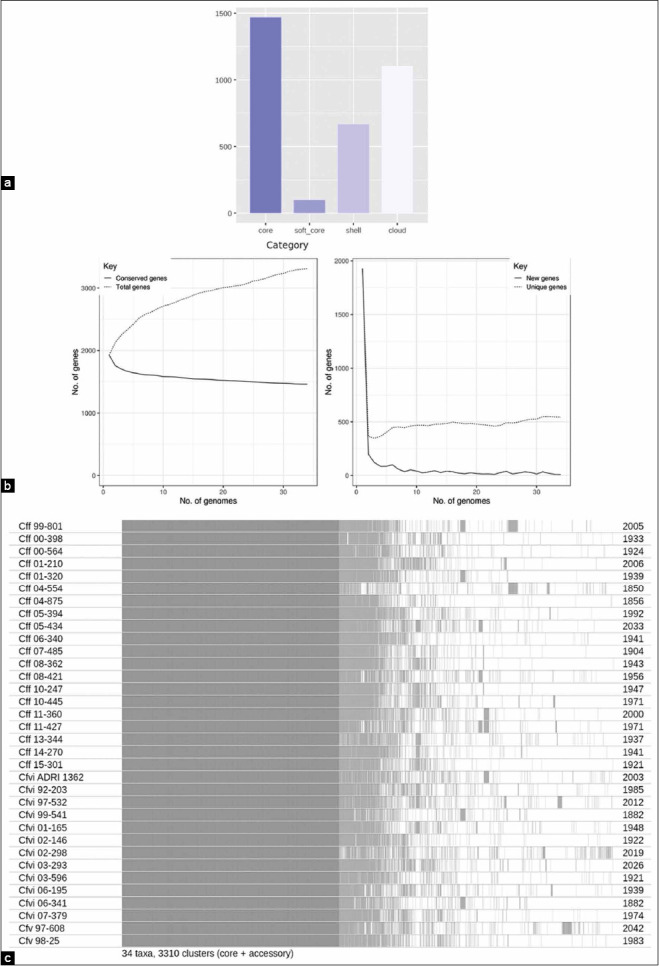
The pangenome of Argentine *Campylobacter fetus* strains. (a) Visualization of the number of genes according to the different categories by Roary [12]. (b) Gene accumulation curve contrasting conserved homologous genes *vs*. total genes in 34 *C. fetus* genomes (left). The curves represent the adjustment of the pangenome size as individual genomes are added (right). (c) Presence-absence profile of genes in each strain. To the right, the number of genes belonging to each isolate.

The present study aimed to get a better understanding about *C. fetus* strains found in Argentina and their counterparts occurring in different parts of the world. For pangenome analysis, whole genome sequences for 34 strains of *C. fetus*, isolated from the cattle in the Pampas region, were obtained from GenBank. In general, the core and the soft-core genes provide information regarding the evolutionary history. In comparison to this, the shell and the cloud genes (which constitute the accessory genome) are involved in lifestyle and adaptation of the organism to different niches. The pangenome analysis for 34 strains showed that 1462 and 1748 genes belonged to the pool of core-genes and accessory genes, respectively (Figure-1a). Figure-1b displays the gene profiles shared between the isolates.

**Figure-2:**
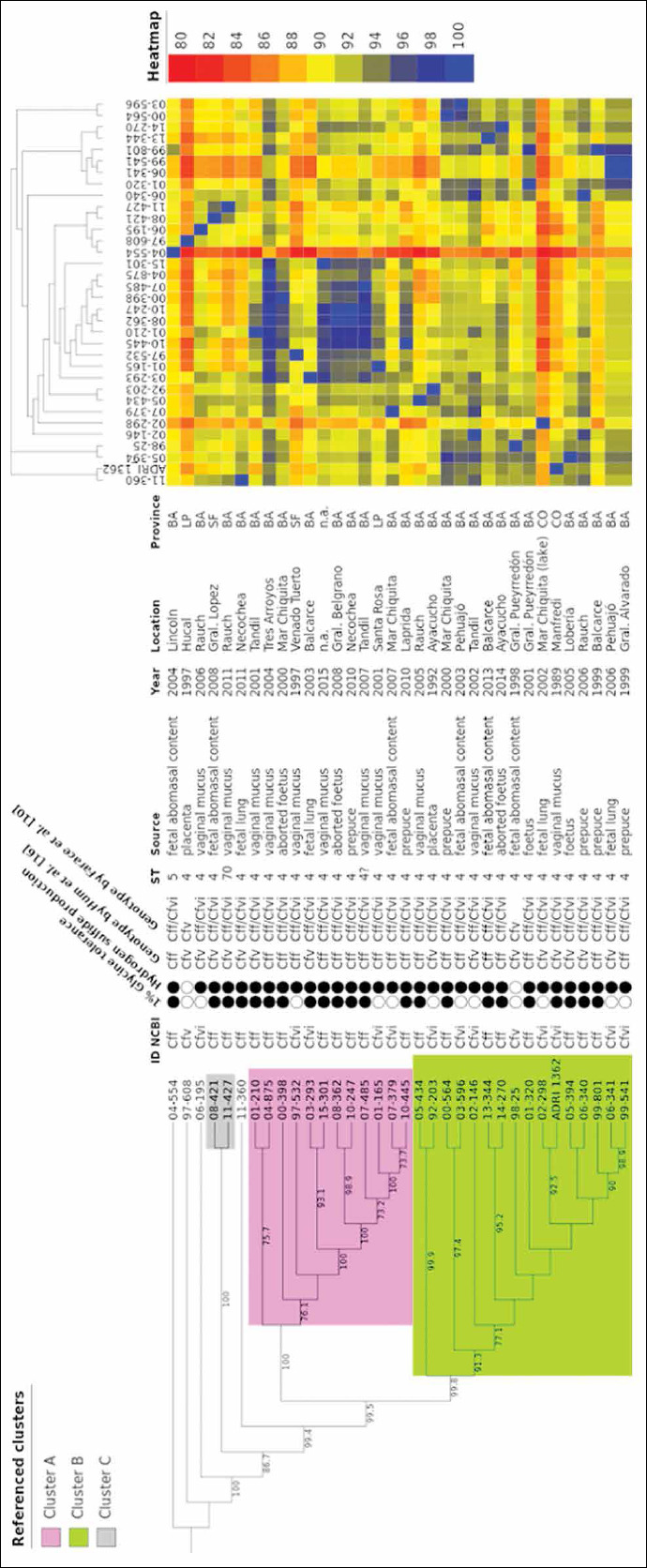
Core genome-based phylogeny and heatmap analysis. Maximum likelihood tree was constructed using IQ-Tree based on 1462 single-copy genes shared by 34 *Campylobacter fetus* strains. The heatmap represents the percentage of shared gene pairs, and is ordered based on a binary tree of presence and absence of accessory genes. The heatmap colors scale from blue (higher percentage of shared genes) to dark red (lower percentage of shared genes). Province codes: BA=Buenos Aires, SF=Santa Fe, CO=Córdoba, LP=La Pampa. n.a: not available.

A heatmap representing the percentage of shared genes was generated using ‘Matrix’ in R Studio. As shown in Figure-2, the strains Cff 04-554 (Buenos Aires), Cfvi 02-298 (Córdoba), and Cfv 97-608 (La Pampa) were characterized by 84.5%, 85.5%, and 86.1%, respectively, of shared genes that were lowest as compared to the rest of the strains. For in-depth analysis of heatmap data, the average percentage of shared genes was calculated by grouping the strains as Cff, Cfv, and Cfvi. Cff, Cfvi and Cfv strains shared individually on average 91.1%, 90.6%, and 88.1% of genes with the complete set of genes of the other two variants, respectively.

No significant differences were detected between the core genomic constitution of subspecies and variants, and these were characterized by significant overall similarity.

### Phylogenetic analysis based on the core genome of *C. fetus*: Local and global analysis

The core genome (1462 genes), built out of 34 *C. fetus* genomes was used to construct a maximum likelihood phylogenetic tree (ML tree) using IQ-Tree. Two major clusters (Cluster A and Cluster B) and a minor cluster (Cluster C) were identified from the ML tree (Figure-2). Among the strains isolated from Argentina, 28 strains were included in the major clusters (A, n=11 and B, n=17), whereas two were grouped into the minor Cluster C. Four strains branched separately from clusters A, B, and C. Interestingly, no clustering was observed among these strains as well. Each cluster included strains from different provinces. Cluster A included strains from Buenos Aires, Santa Fe, and La Pampa. Cluster B included strains isolated from Buenos Aires and Córdoba. Cluster C was associated with strains obtained from Buenos Aires and Santa Fe. Interestingly, Cff 04-554 was found to have lower phylogenetic relationships and it diverged away from the rest of the isolates (Figure-2).

The geographical and temporal distribution of the data for clustering analysis was visualized using Microreact tool. No significant temporal or geographical associations were recorded within the phylogenetic groups. The clustering and metadata (Supplementary Table-1) are available in the following link: https://microreact.org/project/nL5XD1qdA7ALMzgwMXeqiZ.

To investigate the phylogenetic relationships between the strains isolated from Argentina and other parts of the world, a phylogenetic study was conducted using a wide panel of genomic sequences of *C. fetus* strains having distinct origin and isolation source. To ensure consistency in the analysis, the parameters and models used for the local tree were used for global analysis as well. The *C. fetus* core genome built out 265 genomic sequences of strains from 20 countries, encompassed 1143 genes.

**Figure-3:**
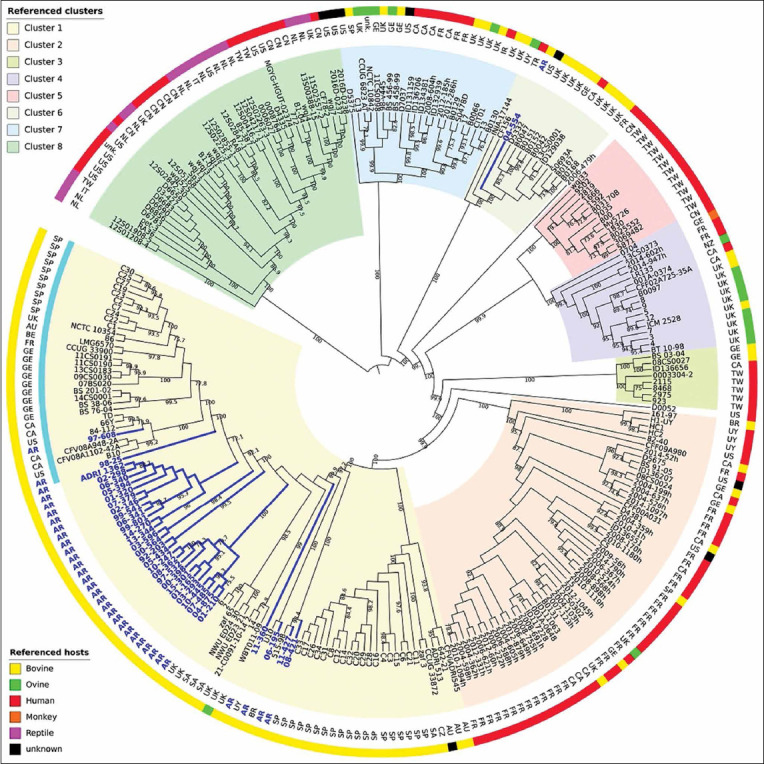
Global phylogeny of *Campylobacter fetus* based on core genome. Maximum likelihood tree based on 1143 single-copy genes shared by 265 *C. fetus* strains. The nodes and clusters colored in blue refer to the Argentine strains (n=34). The turquoise line refers to the subcluster of strains identified as *Campylobacter fetus venerealis* (n=31). Country code: AR=Argentina, AU=Australia, BE=Belgium, BR=Brazil, CA=Canada, CN=China, CZ=Czech Republic, FR=France, GE=Germany, IT=Italy, IR=Ireland, NL=The Netherlands, NZ=New Zealand, SA=South Africa, SP=Spain, TK=Turkey, TW=Taiwan, UK=The United Kingdom, UY=Uruguay, USA=The United States.

The ML tree generated from the analysis of 1143 core genes identified eight well-supported clusters (Figure-3). The major clusters were not geographically defined. A similar phylogenetic tree has been previously reported for a different dataset by Iraola *et al*. [17]. The reptile *C. fetus testudinum* strains diverged from the mammalian *C. fetus* strains (Cluster 8). Among the mammalian clusters (which were the largest one), Cluster 1 corresponded to the “cattle lineage”, a term previously described by Iraola *et al*. [17]. This cluster is exclusive for the strains isolated from cattle. In comparison to this, Clusters 2–7 corresponded to the so called “human lineage” as these were predominated by human isolated strains.

Among 34 strains isolated from the Pampas, 33 strains belonged to Cluster 1. There was one exception, the strain Cff 04-554, which was described as a phylogenetically distant strain in the local analysis. This was further confirmed by the global analysis, wherein Cff 04-554 clustered with nine bovine strains isolated from the United Kingdom and Germany, one ovine strain from Uruguay, and two human strains obtained from Turkey and Canada. Cff 04-554 clustered within Cluster 6, which was nested within the human lineage. Various subclusters could be identified within the cattle-specific Cluster 1. Further analysis revealed the occurrence of certain geographical associations. Most of the strains from Argentina (28/34) exclusively clustered with the strains of same origin. Among the six exceptions, the strains Cff 08-421 and Cff 11-477 shared a minor cluster with a strain isolated from Brazil, while the strain Cfvi 06-195 shared a minor cluster with a strain obtained from Uruguay. Similar to local ML tree analysis, the strain Cff 11-360 did not cluster with any of the strains in global analysis as well.

The remaining two strains, Cfv 97-608 and Cfv 98-25, clustered in a different branch of the subcluster of the strains isolated from Argentina. This minor phylogenetic group included 31 strains having intercontinental distribution. The strain Cfv 97-608 clustered with the strains isolated from Canada and the USA, and the strain Cfv 98-25 formed a singulete. Interestingly, this subcluster included strains identified as Cfv using different typing techniques (Table-1) [10,17-19].

**Table-1:** Description of strains within the putative *Campylobacter fetus venerealis* phylogenetic group.

Strain	Accession number	Year	Origin	Host	Source	ID reported [Ref.]	ID according to L-Cys transporter- PCR [Ref.]
66Y	ERS672211	2012	Canada	Bovine	Prepuce	CFV^a^ [17]	CFV [10]
TD	ERS672212	2011	Canada	Bovine	Prepuce	CFV^a^ [17]	CFV [10]
C1	ERS739275	2009	Spain	Bovine	Prepuce	CFV^a^ [17]	CFV [10]
C2	ERS739276	2007	Spain	Bovine	Prepuce	CFV^a^ [17]	CFV [10]
C7	ERS739281	2007	Spain	Bovine	Prepuce	CFF^a^ [17]	CFV [10]
C19	ERS739293	2006	Spain	Bovine	Prepuce	CFV^a^ [17]	CFV [10]
C22	ERS739296	2008	Spain	Bovine	Prepuce	CFV^a^ [17]	CFV [10]
C23	ERS739297	2007	Spain	Bovine	Prepuce	CFV^a^ [17]	CFV [10]
C24	ERS739298	2010	Spain	Bovine	Prepuce	CFV^a^ [17]	CFV [10]
C25	ERS739299	2011	Spain	Bovine	Prepuce	CFV^a^ [17]	CFV [10]
C27	ERS739301	2011	Spain	Bovine	Prepuce	CFV^a^ [17]	CFV [10]
C30	ERS739304	2014	Spain	Bovine	Prepuce	CFV^a^ [17]	CFV [10]
BS 201/02	ERS686632	2002	Germany	Bovine	Prepuce	CFV^a^ [17]	CFV [10]
BS 76/04	ERS686633	2004	Germany	Bovine	Fetus	CFV^a^ [17]	CFV [10]
BS 38/06	ERS686634	2006	Germany	Bovine	Prepuce	CFV^a^ [17]	CFV [10]
07BS020	ERS686635	2007	Germany	Bovine	Prepuce	CFV^a^ [17]	CFV [10]
09CS0030	ERS686637	2009	Germany	Bovine	Prepuce	CFV^a^ [17]	CFV [10]
11CS0190	ERS686638	2011	Germany	Bovine	Prepuce	CFV^a^ [17]	CFV [10]
11CS0191	ERS686639	2011	Germany	Bovine	Prepuce	CFV^a^ [17]	CFV [10]
13CS0183	ERS686640	2013	Germany	Bovine	Prepuce	CFV^a^ [17]	CFV [10]
14CS0001	ERS686641	2014	Germany	Bovine	Prepuce	CFV^a^ [17]	CFV [10]
97/608	GCA_000759515.1	1997	Argentina	Bovine	Placenta	CFV^a,b^ [10]	CFV [10]
84/112	GCA_000967135.1	1984	United States	Bovine	Vaginal mucus	CFV^a^ [17]	CFV [10]
NCTC 10354	GCA_000222425.1	1952	United Kingdom	Bovine	Vaginal mucus	CFV^a^ [17]	CFV [10]
B6	GCA_000744035.1	1964	Australia	Bovine	Vaginal mucus	CFV^a^ [17]	CFV [10]
B10	LRET00000000	2011	United States	Bovine	unknown	CFV^a,b^ [18]	CFV [10]
CFV08A1102-42A	GCA_011600845.2	2008	Canada	Bovine	Prepuce	CFV^b^ [19]	CFV [This study]
CFV08A948-2A	GCA_011601005.2	2008	Canada	Bovine	Prepuce	CFV^b^ [19]	CFV [This study]
CCUG 33900	LREV00000000	1995	France	Bovine	Abortion	CFV^a,b^ [18]	CFV [10]
LMG 6570	LREW00000000	1985	Belgium	Bovine	unknown	CFV^a,b^ [18]	CFV [10]
98-25	LRES00000000	1998	Argentina	Bovine	Fetus	CFV^a,b^ [10]	CFV [10]

a Molecular typing,

b Biochemical typing

### MLST for the strains isolated from Argentina

*In silico* MLST was performed to obtain the ST for each local *C. fetus* strain. Among 34 local strains, 31 strains were subtyped as ST4. According to the local phylogenetic analysis, ST4 was distributed homogenously among the phylogenetic groups (Figure-2). In accordance with the results obtained for local and global phylogenetic analyses, the strain Cff 04-554 (“human lineage”) was the only strain that showed ST5 (Figures-2 and 3).

Initially, the aforementioned *in silico* approach failed to establish ST for Cff 11-427 and Cff 07-485. In case of the strain Cff 07-485, the *unc*A locus showed low coverage of 85.1%, while the rest of the loci shared 100% nucleotide identity with ST4. The genome sequence analysis for Cff 11-427 showed the presence of a C-to-T transition at position 293 of the *unc*A allele. This new allele and its respective ST were deposited in the PubMLST database (http://pubmlst.org). This strain was assigned *unc*A allelic variant 15 and ST70 subtype. This ST70 strain clustered with a ST4 strain (Figure-2). This variant has been reported for the 1^st^ time in the present study.

## Discussion

In the present study, local phylogenetic analysis of 34 *C. fetus* strains, isolated from the Pampas region, was performed. The study allowed a critical evaluation of the existence of regional variants to describe the circulating *C. fetus* strains in Argentina. The results of the analysis showed clustering of most of the strains into few clusters, suggesting a clonal population structure where two major overlapping clusters were identified in Argentina. These two major clusters included 82% of the local strains.

The use of Microreact software allowed an interactive visualization of the dynamics of isolation in terms of year, frequency of geographical distribution, and source of isolation for each of the *C. fetus* strains included in the local study. The clustering data showed no association with the source of the samples, date of isolation, origin, and biochemical tests. The two strains that were included in the minor cluster were isolated at two different time points, 1997 and 2008. In fact, these strains were isolated from two distinct provinces, Buenos Aires and Santa Fe, from localities that were 500 km away. On the other hand, the second strain isolated from Santa Fe belonged to the major Cluster A. To establish the existence of a putative third cluster for the distribution of the strains throughout the region, the analysis must include a large sample pool. Interestingly, Córdoba Province was under represented and only two strains from this region were studied. These strains formed a subcluster within the major Cluster B. Similarly, two strains isolated in La Pampa Province were included in the study. However, one of these strains clustered in the major Cluster A, while the other one was found to be phylogenetically distant.

The clonal nature of *C. fetus* might be attributed to higher genetic stability of this pathogen as compared to other *Campylobacter* species [20,21]. Thus, there is a significant possibility for the continuous circulation of few genotypes of *C. fetus* in this endemic region of Argentina. Such circulation might be indicative of the movement of cattle over time or trade of livestock material between different regions. In ­addition, different variants were observed within the dominant clones. This might be attributed to the use of different herd management practices (like vaccination) or different cattle breeds, which could have driven the selection of the strains.

The global phylogenetic analysis provided a broad overview of the relationships between the strains obtained from Argentina and their global counterparts that were isolated from different hosts in different countries. In a recent study, Iraola *et al*. [17] described the phylogeny of *C. fetus*. The study proposed two major lineages for *C. fetus* strains, human, and cattle lineage . In the present study, 33 out of 34 strains, isolated from Argentina, belonged to the cattle lineage. The global ML tree was consistent with the findings of the local ML tree. It was successful in clarifying the position of the phylogenetically distant strains. The bovine strain Cff 04-554, which belonged to the human lineage, showed higher genetic distance in the clustering. In addition to this, it was associated with significant differences at the core genome level. The genome sequence of this strain was manually checked to avoid any issues arising due to chimeras or other assembly artifacts. The comparative analyses of the genomes showed the presence of large number of polymorphisms in this strain, including SNPs and insertions in some of the genes.

Cff 04-554 strain was isolated from a 7.5-month-old aborted fetus. This fetus belonged to a herd where both artificial and natural insemination was practiced in cows. Among the various strains obtained from Argentina, this strain was unique and it was assigned to ST5 according to the PubMLST database. ST5 subtype has been previously reported in cattle and human strains of *C. fetus* isolated from different countries such as the United States of America, Belgium, Germany, and the United Kingdom [20]. Interestingly, Cff 04-554 clustered with these previously reported strains. An explanation regarding the presence of this strain in the livestock productive system of Argentina remains elusive; however, the existence of international trade for livestock (semen, embryos, or even animals) in the past could not be ignored. In addition, this strain also clustered with one ovine strain obtained from Uruguay [22]. Thus, there is possibility for the circulation of this genotype in different hosts in South America. More studies are required to test the prevalence and relevance of this genotype.

Several previous studies have proposed the suitability of Pulsed Field Gel Electrophoresis (PFGE), Amplified Fragment Length Polymorphism (AFLP), and MLST as genotyping tools for subtyping of *C. fetus* strains. PFGE and AFLP have been successfully utilized for subspecies differentiation but have not been extensively studied [23,24]. In comparison to these, MLST is an unambiguous and less complex procedure, which is based on the sequencing of housekeeping genes. This technique is robust and the sequencing data can be compared with the help of open access database. Thus, MLST has gained wide acceptance for the evaluation of *C. fetus* diversity in the past few years promoted in large part by the sequencing costs reduction. In a previous study, MLST showed low inter-ST genetic diversity and the two subspecies of *C. fetus* were described to have close genetic relation. The study suggested the suitability of MLST for long-term epidemiological and phylogenetic analyses [20]. In another study, MLST results were in concordance with the core genome clustering. These results further suggested that the loci included in the MLST scheme represent a suitable subset of genes of the core genome [18].

In concordance with the phylogenetic analysis, MLST results revealed low diversity among *C. fetus* strains isolated from the Pampas region. ST4 was found to be most common subtype, with inclusion of 31 strains. One strain belonged to ST5, while another one was identified as a new variant and was designated a new subtype, ST70. However, this technique failed to efficiently discriminate between the strains located in the major clusters. In addition to this, ST4 was associated with all the clusters. Interestingly, ST5 and ST70 strains were grouped outside the major clusters. Initially, ST4 was first found to be exclusively associated with cattle Cfv strains [20]. Thus, ST4 was proposed to be cattle-associated genotype. However, later, Iraola *et al*. [25] identified an ST4 Cff strain in a rural worker, representing a probable case of zoonotic transmission. The study also reported inconsistencies between MLST and whole-genome typing outcomes. In concordance with the findings of Iraola *et al*. [25], MLST analysis in the present study was found to be partially concordant with phylogenetic analysis. Thus, all these observations suggested the limited utility of MLST as a tool to evaluate the genetic diversity of circulating *C. fetus* strains in Argentina.

NGS and phylogenetic studies have provided significant information about this pathogen; however, subspecies assignment and their differential diagnosis remain a great challenge. In the present study, an approach similar to the one used by Iraola *et al*. [17] was followed, which resulted in same tree topology and clustering. Interestingly, a particular sub-branch of 31 strains with common characteristics was identified within the cattle lineage. In a previous study, Farace *et al*. reported the use of a Polymerase chain reaction (PCR)-based testing method to evaluate the presence of a cysteine transporter operon (L-Cys transporter) linked to hydrogen sulfide production in *C. fetus* strains [10]. All the strains from this subcluster were tested by *in silico*-PCR and were identified as non-producers for hydrogen sulfide, a trait typical of Cfv. Additionally, this analysis was consistent with the biochemical results obtained for the strains included in this subcluster. Most of these strains were isolated from preputial samples (71%). Bulls’ preputial crypts have been previously described as the main niche for the existence of this subspecies [26]. In addition to this, most of these strains shared molecular traits and were identified as Cfv through molecular typing methods. Among these, the Spanish strain C7 was the only exception. This strain has been molecularly typed as Cff by Iraola *et al*. [17]. In comparison to this, Farace *et al*. described it as Cfv on the basis of PCR-based testing for L-Cys transporter [10]. Cfv 98-25, isolated from Argentina, has been associated with conflicting biochemical classifications in different labs [10]. However, in the present study, this strain was biochemically typed as Cfv, which was consistent with the results for L-Cys transporter-PCR, conventional molecular typing [16], and phylogenetic analysis. Interestingly, the rest of the strains within the cattle lineage were found to be hydrogen sulfide-producing strains, a characteristic common to both Cff and Cfv biovar intermedius strains.

In a previous study, van der Graaf-van Bloois *et al*. [18] performed core genome phylogenetic analysis for 21 *C. fetus* genome sequences. The clustering results for the study were found to be inconsistent with the phenotype of the strains. The discrepancies in the results for the present study and the study by van der Graaf-van Bloois *et al*. [18] could be attributed to the composition of the genomic dataset. Significantly different sample size was used in both studies, which might have a significant impact on the core genome constitution and clustering. It is important to mention that no evidences have been reported for any correlation between hydrogen sulfide production and virulence of the strain. Thus, the results of the present study should be interpreted with caution. For future analyses, well-characterized virulence markers must be used. There are many questions that still remain unanswered, particularly regarding the importance of each subspecies in cattle health and differential diagnosis. Several interesting points, like whether these subspecies are actually genetically distinct and do enough evidence exist to confirm (or deny) the importance of one subspecies over the other, should be discussed. The subspecies assignment should be upheld by both phenotypic and molecular evidences and must also be supported by phylogenetic analysis. In the present study, phylogenetic analysis was successful in differentiating a subset of strains that shared Cfv phenotypic and genotypic traits, and future studies should further evaluate its relevance in cattle health.

Despite the use of low number of samples from Argentina, the present study provided basic information that would assist in the designing of future epidemiological studies to get better insights into *C. fetus* related infections in the country. Argentina (2,780,400 km^2^) contains different phytogeographic regions where livestock is less important as compared to the Pampas region. However, livestock production is relevant for the local economies and reproductive diseases are also prevalent. In these regions, the diagnosis is frequently based on techniques that do not involve isolation of *C. fetus*. This has particularly delayed the evaluation of circulating strains in Argentina. Further phylogenetic studies, particularly focused on the inclusion of under sampled regions and augmentation of overall number of samples, might be helpful in providing a more realistic overview regarding the phylogenetic relationships of *C. fetus* strains isolated from Argentina. The present work focused largely on the analysis of genomic data of *C. fetus* strains isolated from cattle in the Pampas region, the most productive area of Argentina. However, environmental samples and human strains must also be studied for better understanding. The outcomes of the present study encourage the sharing of data for strains isolated from different sources to expand the knowledge for this pathogen in Argentina.

## Conclusion

Local and global phylogenomic analyses revealed the circulation of a limited number of *C. fetus* strains in Argentina over the years. The results also suggested an active movement of animals, probably due to economic trade between the different regions of the country as well as with neighboring countries such as Brazil and Uruguay. Although the results for MLST showed partial concordance with the phylogenetic analysis, MLST failed to qualify as a reliable subtyping method to assess *C. fetus* diversity in Argentina. The study provided significant background genomic information and updated metadata, which can be further, used as a platform for future surveillance and tracking of *C. fetus* distribution in Argentina.

## Authors’ Contributions

AKG and AFA conceived and supervised the study. PDF and JMI designed the study, collected and analyzed genomic data. FAP, CGM, MAM and JAG collected metadata. AKG, PDF, FAP, CGM, and JAG interpreted the results. AKG and PDF drafted the manuscript. All the authors read and approved the final version of the manuscript.

## Supplementary Tables

**Supplementary Table-1:** Metadata of Argentine *C. fetus* strains.

Strain	Accession Number	Year	1% glycine tolerance	H_2_S production	Phenotype	Genotype		Source	District (Province)	MLST-allele							ST
	
						Hum *et al*. [16]	Farace *et al*. [10]			*asp*A	*gln*A	*glt*A	*gly*A	*pgm*	*tkt*	*unc*A	
99-801	ERS739235	1999	+	+	Cff	Cff	Cff/Cfvi	Prepuce	Balcarce (Buenos Aires)	1	2	2	2	1	2	1	4
00-398	ERS739236	2000	+	+	Cff	Cff	Cff/Cfvi	Aborted fetus	Mar Chiquita (Buenos Aires)	1	2	2	2	1	2	1	4
00-564	ERS739237	2000	+	+	Cff	Cff	Cff/Cfvi	Prepuce	Mar Chiquita (Buenos Aires)	1	2	2	2	1	2	1	4
01-210	ERS739239	2001	+	+	Cff	Cfv	Cff/Cfvi	Vaginal mucus	Tandil (Buenos Aires)	1	2	2	2	1	2	1	4
01-320	ERS739238	2001	+	+	Cff	Cff	Cff/Cfvi	Fetus	Gral. Pueyrredón (Buenos Aires)	1	2	2	2	1	2	1	4
04-554	CP008808-CP008809	2004	+	+	Cff	Cff	Cff/Cfvi	Fetal abomasal content	Lincoln (Buenos Aires)	1	1	1	2	1	1	3	5
04-875	ERS739242	2004	+	+	Cff	Cff	Cff/Cfvi	Vaginal mucus	Tres Arroyos (Buenos Aires)	1	2	2	2	1	2	1	4
05-394	ERS739243	2005	+	+	Cff	Cfv	Cff/Cfvi	Fetus	Lobería (Buenos Aires)	1	2	2	2	1	2	1	4
05-434	ERS739244	2005	+	+	Cff	Cff	Cff/Cfvi	Vaginal mucus	Rauch (Buenos Aires)	1	2	2	2	1	2	1	4
06-340	ERS739245	2006	+	+	Cff	Cfv	Cff/Cfvi	Prepuce	Rauch (Buenos Aires)	1	2	2	2	1	2	1	4
07-485	ERS739248	2007	+	+	Cff	Cff	Cff/Cfvi	Vaginal mucus	Tandil (Buenos Aires)	1?	2	2	2	1	2	1	4?
08-362	ERS739249	2008	+	+	Cff	Cff	Cff/Cfvi	Aborted fetus	Gral. Belgrano (Buenos Aires)	1	2	2	2	1	2	1	4
08-421	SOOT00000000	2008	+	+	Cff	Cfv	Cff/Cfvi	Fetal abomasal content	Gral. López (Santa Fe)	1	2	2	2	1	2	1	4
10-247	ERS739250	2010	+	+	Cff	Cff	Cff/Cfvi	Prepuce	Necochea (Buenos Aires)	1	2	2	2	1	2	1	4
10-445	ERS739251	2010	+	+	Cff	Cff	Cff/Cfvi	Prepuce	Laprida (Buenos Aires)	1	2	2	2	1	2	1	4
11-360	ERS739252	2011	+	+	Cff	Cfv	Cff/Cfvi	Fetal lung	Necochea (Buenos Aires)	1	2	2	2	1	2	1	4
11-427	ERS739253	2011	+	+	Cff	Cfv	Cff/Cfvi	Vaginal mucus	Rauch (Buenos Aires)	1	2	2	2	1	2	15	70
13-344	SOYX00000000	2013	+	+	Cff	Cff	Cff/Cfvi	Fetal abomasal content	Balcarce (Buenos Aires)	1	2	2	2	1	2	1	4
14-270	ERS739254	2014	+	+	Cff	Cff	Cff/Cfvi	Aborted fetus	Ayacucho (Buenos Aires)	1	2	2	2	1	2	1	4
15-301	ERS739255	2015	+	+	Cff	Cff	Cff/Cfvi	Vaginal mucus	n.a.	1	2	2	2	1	2	1	4
97-608	CP008810-CP008812	1997	-	-	Cfv	Cfv	Cfv	Placenta	Hucal (La Pampa)	1	2	2	2	1	2	1	4
ADRI 1362	LREX00000000	1989	+	+	Cff	Cfv	Cff/Cfvi	Vaginal mucus	Manfredi (Córdoba)	1	2	2	2	1	2	1	4
92-203	LRVL00000000	1992	-	+	Cfvi	Cfv	Cff/Cfvi	Placenta	Ayacucho (Buenos Aires)	1	2	2	2	1	2	1	4
97-532	LRER00000000	1997	-	+	Cfvi	Cff	Cff/Cfvi	Vaginal mucus	Venado Tuerto (Santa Fe)	1	2	2	2	1	2	1	4
98-25	LRES00000000	1998	-	-	Cfv	Cfv	Cfv	Fetal abomasal content	Gral. Pueyrredón (Buenos Aires)	1	2	2	2	1	2	1	4
99-541	ASTK00000000	1999	-	+	Cfvi	Cff	Cff/Cfvi	Prepuce	Gral. Alvarado (Buenos Aires)	1	2	2	2	1	2	1	4
01-165	CP014568-CP014570	2001	-	+	Cfvi	Cff	Cff/Cfvi	Vaginal mucus	Santa Rosa (La Pampa)	1	2	2	2	1	2	1	4
02-146	ERS739240	2002	-	+	Cfvi	Cfv	Cff/Cfvi	Vaginal mucus	Tandil (Buenos Aires)	1	2	2	2	1	2	1	4
02-298	LRVK00000000	2002	-	+	Cfvi	Cfv	Cff/Cfvi	Fetal lung	Mar Chiquita (lake) (Córdoba)	1	2	2	2	1	2	1	4
03-293	CP0006999-CP007002	2003	+	+	Cff	Cfv	Cff/Cfvi	Fetal lung	Balcarce (Buenos Aires)	1	2	2	2	1	2	1	4
03-596	LRAM00000000	2003	-	+	Cfvi	Cff	Cff/Cfvi	Fetal abomasal content	Pehuajó (Buenos Aires)	1	2	2	2	1	2	1	4
06-195	ERS739246	2006	-	+	Cfvi	Cfv	Cff/Cfvi	Vaginal mucus	Rauch (Buenos Aires)	1	2	2	2	1	2	1	4
06-341	SOYW00000000	2006	-	+	Cfvi	Cfv	Cff/Cfvi	Fetal lung	Pehuajó (Buenos Aires)	1	2	2	2	1	2	1	4
07-379	ERS739247	2007	-	+	Cfvi	Cff	Cff/Cfvi	Fetal abomasal content	Mar Chiquita (Buenos Aires)	1	2	2	2	1	2	1	4

? Cfe_aspA and ST: alleles with <100% coverage found. Uncertain hit, ST cannot be trusted. n.a=Not available, *C. fetus=Campylobacter fetus*

**Supplementary Table-2:** Global dataset and in silico L-Cys transporter-PCR results.

Strain	Accession number	Host	Source	Origin	ID according to L-Cys transporter-PCR [Ref.]
00-398	ERS739236	Bovine	Aborted fetus	Argentina	CFF/CFVI [10]
00-564	ERS739237	Bovine	Prepuce	Argentina	CFF/CFVI [10]
01-165	CP014568-CP014570	Bovine	Vaginal mucus	Argentina	CFF/CFVI [10]
01-210	ERS739239	Bovine	Vaginal mucus	Argentina	CFF/CFVI [10]
01-320	ERS739238	Bovine	Fetus	Argentina	CFF/CFVI [10]
02-146	ERS739240	Bovine	Vaginal mucus	Argentina	CFF/CFVI [10]
02-298	GCA_001699555.1	Bovine	Fetal lung	Argentina	CFF/CFVI [10]
03-293	CP0006999-CP007002	Bovine	Fetal lung	Argentina	CFF/CFVI [10]
03-596	LRAM00000000	Bovine	Fetal abomasal content	Argentina	CFF/CFVI [10]
04-875	ERS739242	Bovine	Vaginal mucus	Argentina	CFF/CFVI [10]
05-394	ERS739243	Bovine	Fetus	Argentina	CFF/CFVI [10]
05-434	ERS739244	Bovine	Vaginal mucus	Argentina	CFF/CFVI [10]
06-195	ERS739246	Bovine	Vaginal mucus	Argentina	CFF/CFVI [10]
06-340	ERS739245	Bovine	Prepuce	Argentina	CFF/CFVI [10]
06-341	SOYW00000000	Bovine	Fetal lung	Argentina	CFF/CFVI [10]
07-379	ERS739247	Bovine	Fetal abomasal content	Argentina	CFF/CFVI [10]
07-485	ERS739248	Bovine	Vaginal mucus	Argentina	CFF/CFVI [10]
08-362	ERS739249	Bovine	Aborted fetus	Argentina	CFF/CFVI [10]
08-421	SOOT00000000	Bovine	Fetal abomasal content	Argentina	CFF/CFVI [10]
10-247	ERS739250	Bovine	Prepuce	Argentina	CFF/CFVI [10]
10-445	ERS739251	Bovine	Prepuce	Argentina	CFF/CFVI [10]
11-360	ERS739252	Bovine	Fetal lung	Argentina	CFF/CFVI [10]
11-427	ERS739253	Bovine	Vaginal mucus	Argentina	CFF/CFVI [10]
13-344	SOYX00000000	Bovine	Fetal abomasal content	Argentina	CFF/CFVI [10]
14-270	ERS739254	Bovine	Aborted fetus	Argentina	CFF/CFVI [10]
15-301	ERS739255	Bovine	Vaginal mucus	Argentina	CFF/CFVI [10]
92-203	LRVL00000000	Bovine	Placenta	Argentina	CFF/CFVI [10]
97-532	LRER00000000	Bovine	Vaginal mucus	Argentina	CFF/CFVI [10]
99-541	ASTK00000000	Bovine	Prepuce	Argentina	CFF/CFVI [10]
99-801	ERS739235	Bovine	Prepuce	Argentina	CFF/CFVI [10]
ADRI 1362	LREX00000000	Bovine	Vaginal mucus	Argentina	CFF/CFVI [10]
04-554	CP008808-CP008809	Bovine	Fetal abomasal content	Argentina	CFF/CFVI [10]
642-21	AJSG00000000	Bovine	Uterus	Australia	CFF/CFVI [10]
ADRI 513	LRFA00000000	unknown	Unknown	Australia	CFF/CFVI [10]
161-97	ERS846568	Bovine	Prepuce	Brazil	CFF/CFVI [10]
515-98	ERS846569	Bovine	Prepuce	Brazil	CFF/CFVI [10]
001A-0374	ERS686652	Human	Blood	Canada	CFF/CFVI [10]
001A-0648	ERS686653	Human	Blood	Canada	CFF/CFVI [10]
ID111063	ERS739225	Human	Blood	Canada	CFF/CFVI [10]
ID117228	ERS739226	Human	Blood	Canada	CFF/CFVI [10]
ID129038	ERS739227	Human	Blood	Canada	CFF/CFVI [10]
ID131159	ERS739228	Human	Feces	Canada	CFF/CFVI [10]
ID132939	ERS739234	Human	Blood	Canada	CFF/CFVI [10]
ID134381	ERS739229	Human	Feces	Canada	CFF/CFVI [10]
ID136207	ERS739230	Human	Blood	Canada	CFF/CFVI [10]
ID136551	ERS739231	Human	Blood	Canada	CFF/CFVI [10]
ID136656	ERS739232	Human	Blood	Canada	CFF/CFVI [10]
ID136706	ERS739233	Human	Blood	Canada	CFF/CFVI [10]
CCUG 33872	LREU00000000	Bovine	Abortion	Czech Republic	CFF/CFVI [10]
2004-103h	ERS672233	Human	Cerebrospinal fluid	France	CFF/CFVI [10]
2004-199h	ERS672234	Human	Cerebrospinal fluid	France	CFF/CFVI [10]
2004-359h	ERS672235	Human	Blood	France	CFF/CFVI [10]
2004-362h	ERS672236	Human	Placenta	France	CFF/CFVI [10]
2004-526h	ERS672237	Human	Feces	France	CFF/CFVI [10]
2004-598h	ERS672238	Human	Blood	France	CFF/CFVI [10]
2004-605h	ERS672239	Human	Feces	France	CFF/CFVI [10]
2004-637h	ERS672240	Human	Joint fluid	France	CFF/CFVI [10]
2006-222h	ERS672241	Human	Blood	France	CFF/CFVI [10]
2006-367h	ERS672242	Human	Cerebrospinal fluid	France	CFF/CFVI [10]
2006-479h	ERS672243	Human	Feces	France	CFF/CFVI [10]
2006-588h	ERS672244	Human	Cerebrospinal fluid	France	CFF/CFVI [10]
2006-621h	ERS672245	Human	Blood	France	CFF/CFVI [10]
2006-649h	ERS672246	Human	Feces	France	CFF/CFVI [10]
2007-123h	ERS672271	Human	Cerebrospinal fluid	France	CFF/CFVI [10]
2008-170h	ERS672247	Human	Feces	France	CFF/CFVI [10]
2008-568h	ERS672248	Human	Joint fluid	France	CFF/CFVI [10]
2008-604h	ERS672249	Human	Feces	France	CFF/CFVI [10]
2008-691h	ERS672250	Human	Cerebrospinal fluid	France	CFF/CFVI [10]
2008-755h	ERS672251	Human	Blood	France	CFF/CFVI [10]
2008-898h	ERS672252	Human	Blood	France	CFF/CFVI [10]
2009-56h	ERS672272	Human	Cerebrospinal fluid	France	CFF/CFVI [10]
2010-1094h	ERS672255	Human	Blood	France	CFF/CFVI [10]
2010-1119h	ERS672256	Human	Feces	France	CFF/CFVI [10]
2010-1180h	ERS672257	Human	Blood	France	CFF/CFVI [10]
2010-41h	ERS672253	Human	Feces	France	CFF/CFVI [10]
2010-524h	ERS672254	Human	Kidney	France	CFF/CFVI [10]
2012-1045h	ERS672264	Human	Joint fluid	France	CFF/CFVI [10]
2012-185h	ERS672259	Human	Blood	France	CFF/CFVI [10]
2012-286h	ERS672260	Human	Blood	France	CFF/CFVI [10]
2012-331h	ERS672261	Human	Blood	France	CFF/CFVI [10]
2012-60h	ERS672258	Human	Feces	France	CFF/CFVI [10]
2012-879h	ERS672263	Human	Feces	France	CFF/CFVI [10]
2014-1097h	ERS672270	Human	Feces	France	CFF/CFVI [10]
2014-52h	ERS672265	Human	Cerebrospinal fluid	France	CFF/CFVI [10]
2014-602h	ERS672266	Human	Blood	France	CFF/CFVI [10]
2014-790h	ERS672267	Human	Blood	France	CFF/CFVI [10]
2014-947h	ERS672269	Human	Blood	France	CFF/CFVI [10]
08CS0024	ERS686636	Bovine	Prepuce	Germany	CFF/CFVI [10]
08CS0027	ERS686646	Bovine	Prepuce	Germany	CFF/CFVI [10]
11CS0098	ERS686648	Ovine	Placenta	Germany	CFF/CFVI [10]
12CS0302	ERS686649	Bovine	Prepuce	Germany	CFF/CFVI [10]
13CS0001	ERS686650	Bovine	Prepuce	Germany	CFF/CFVI [10]
13CS0373	ERS686651	Monkey	Feces	Germany	CFF/CFVI [10]
BS 03-04	ERS686644	Bovine	Fetus	Germany	CFF/CFVI [10]
BS 456-99	ERS686642	Ovine	Fetus	Germany	CFF/CFVI [10]
BS 458-99	ERS686643	Bovine	Fetus	Germany	CFF/CFVI [10]
BS 91-05	ERS686645	Bovine	Prepuce	Germany	CFF/CFVI [10]
CIT01	RBHV00000000	Human	Peripheral blood culture	Ireland	CFF/CFVI [10]
LR133	ERS846544	Ovine	Fetus	New Zealand	CFF/CFVI [10]
zaf3	LREZ00000000	Bovine	Fetus	South Africa	CFF/CFVI [10]
zaf65	LREY00000000	Bovine	Unknown	South Africa	CFF/CFVI [10]
C11	ERS739285	Bovine	Prepuce	Spain	CFF/CFVI [10]
C12	ERS739286	Bovine	Prepuce	Spain	CFF/CFVI [10]
C13	ERS739287	Bovine	Prepuce	Spain	CFF/CFVI [10]
C14	ERS739288	Bovine	Prepuce	Spain	CFF/CFVI [10]
C15	ERS739289	Bovine	Prepuce	Spain	CFF/CFVI [10]
C16	ERS739290	Bovine	Prepuce	Spain	CFF/CFVI [10]
C17	ERS739291	Bovine	Prepuce	Spain	CFF/CFVI [10]
C20	ERS739294	Bovine	Prepuce	Spain	CFF/CFVI [10]
C21	ERS739295	Bovine	Prepuce	Spain	CFF/CFVI [10]
C26	ERS739300	Bovine	Prepuce	Spain	CFF/CFVI [10]
C28	ERS739302	Bovine	Prepuce	Spain	CFF/CFVI [10]
C29	ERS739303	Bovine	Prepuce	Spain	CFF/CFVI [10]
C3	ERS739277	Bovine	Prepuce	Spain	CFF/CFVI [10]
C31	ERS739305	Bovine	Prepuce	Spain	CFF/CFVI [10]
C32	ERS739306	Bovine	Prepuce	Spain	CFF/CFVI [10]
C33	ERS739307	Bovine	Prepuce	Spain	CFF/CFVI [10]
C34	ERS739308	Bovine	Prepuce	Spain	CFF/CFVI [10]
C4	ERS739278	Bovine	Prepuce	Spain	CFF/CFVI [10]
C5	ERS739279	Bovine	Prepuce	Spain	CFF/CFVI [10]
C6	ERS739280	Bovine	Prepuce	Spain	CFF/CFVI [10]
C8	ERS739282	Bovine	Prepuce	Spain	CFF/CFVI [10]
800	ERS739271	Human	Blood	Taiwan	CFF/CFVI [10]
923	ERS739257	Human	Blood	Taiwan	CFF/CFVI [10]
1592	ERS739260	Human	Blood	Taiwan	CFF/CFVI [10]
1666	ERS739267	Human	Blood	Taiwan	CFF/CFVI [10]
1830	ERS739261	Human	Blood	Taiwan	CFF/CFVI [10]
2115	ERS739264	Human	Blood	Taiwan	CFF/CFVI [10]
2819	ERS739265	Human	Blood	Taiwan	CFF/CFVI [10]
2975	ERS739256	Human	Blood	Taiwan	CFF/CFVI [10]
5871	ERS739266	Human	Blood	Taiwan	CFF/CFVI [10]
7035	ERS739258	Human	Blood	Taiwan	CFF/CFVI [10]
8468	ERS739262	Human	Blood	Taiwan	CFF/CFVI [10]
9502	ERS739270	Human	Blood	Taiwan	CFF/CFVI [10]
3069482	ERS739274	Human	Blood	Taiwan	CFF/CFVI [10]
8025552	ERS739273	Human	Blood	Taiwan	CFF/CFVI [10]
8031708	ERS739272	Human	Blood	Taiwan	CFF/CFVI [10]
0003304-2	ERS739263	Human	Blood	Taiwan	CFF/CFVI [10]
My5726	ERS739259	Human	Blood	Taiwan	CFF/CFVI [10]
CF156	ERS672273	Human	Blood	Turkey	CFF/CFVI [10]
1	ERS846553	Bovine	Prepuce	United Kingdom	CFF/CFVI [10]
2	ERS846554	Bovine	Prepuce	United Kingdom	CFF/CFVI [10]
3	ERS846555	Ovine	Placenta	United Kingdom	CFF/CFVI [10]
4	ERS846556	Ovine	Placenta	United Kingdom	CFF/CFVI [10]
5	ERS846557	Ovine	Placenta	United Kingdom	CFF/CFVI [10]
6	ERS846558	Bovine	Prepuce	United Kingdom	CFF/CFVI [10]
7	ERS846559	Ovine	Fetus	United Kingdom	CFF/CFVI [10]
8	ERS846560	Ovine	Fetus	United Kingdom	CFF/CFVI [10]
9	ERS846561	Ovine	Placenta	United Kingdom	CFF/CFVI [10]
12	ERS846562	Ovine	Placenta	United Kingdom	CFF/CFVI [10]
13	ERS846563	Bovine	Prepuce	United Kingdom	CFF/CFVI [10]
14	ERS846564	Ovine	Placenta	United Kingdom	CFF/CFVI [10]
15	ERS846565	Ovine	Placenta	United Kingdom	CFF/CFVI [10]
17	ERS846566	Ovine	Fetus	United Kingdom	CFF/CFVI [10]
21-C0091-10-14_2	ERS672276	Bovine	Prepuce	United Kingdom	CFF/CFVI [10]
98-v445	LMBH00000000	Bovine	Bull	United Kingdom	CFF/CFVI [10]
B0042	ERR419595	Bovine	Feces	United Kingdom	CFF/CFVI [10]
B0047	ERR419600	Bovine	Feces	United Kingdom	CFF/CFVI [10]
B0066	ERR419653	Bovine	Feces	United Kingdom	CFF/CFVI [10]
B0097	ERR419653	Bovine	Feces	United Kingdom	CFF/CFVI [10]
B0129	ERR419637	Bovine	Feces	United Kingdom	CFF/CFVI [10]
B0130	ERR419638	Bovine	Feces	United Kingdom	CFF/CFVI [10]
B0131	ERR419639	Bovine	Feces	United Kingdom	CFF/CFVI [10]
B0151	ERR419648	Bovine	Feces	United Kingdom	CFF/CFVI [10]
B0152	ERR419649	Bovine	Feces	United Kingdom	CFF/CFVI [10]
B0167	ERR460866	Bovine	Feces	United Kingdom	CFF/CFVI [10]
B0168	ERR460867	Bovine	Feces	United Kingdom	CFF/CFVI [10]
BT 10-98	LRAL00000000	Ovine	Unknown	United Kingdom	CFF/CFVI [10]
JCM_2528	ERS846567	Bovine	Vaginal mucus	United Kingdom	CFF/CFVI [10]
S0478D	ERR419653	Bovine	Feces	United Kingdom	CFF/CFVI [10]
S0693A	ERR419284	Bovine	Feces	United Kingdom	CFF/CFVI [10]
WBT 011-09	LMBI00000000	Bovine	Unknown	United Kingdom	CFF/CFVI [10]
82-40	CP000487	Human	Blood	United States	CFF/CFVI [10]
NCTC 10842	LS483431	Ovine	Unknown	Unknown	CFF/CFVI [10]
H1-UY	JYCP00000000	Human	Blood	Uruguay	CFF/CFVI [10]
HC1	QJTR00000000	Human	Blood	Uruguay	CFF/CFVI [10]
HC2	QJTS00000000	Human	Cerebrospinal fluid	Uruguay	CFF/CFVI [10]
CFViADRI545	GCA_011601375.2	Bovine	Reproductive tract	Australia	CFF/CFVI [this study]
CFF00A031	GCA_011600945.2	Bovine	Prepuce	Canada	CFF/CFVI [this study]
CFF02A725-35A	GCA_011600855.2	Bovine	Prepuce	Canada	CFF/CFVI [this study]
CFF09A980	GCA_011600995.2	Bovine	Prepuce	Canada	CFF/CFVI [this study]
0704	GCA_010120585.1	Human	Ascites	China	CFF/CFVI [this study]
wqj33	GCA_001699735.1	Human	Blood	China	CFF/CFVI [this study]
NWU_ED23_21	GCA_013406955.1	Bovine	Unknown	South Africa	CFF/CFVI [this study]
NWU_ED24_30	GCA_013406925.1	Bovine	Unknown	South Africa	CFF/CFVI [this study]
CCUG_6823_AT	GCA_008693125.1	Ovine	Fetus brain	United Kingdom	CFF/CFVI [this study]
D0052	GCA_008014295.1	Human	Abscess	United States	CFF/CFVI [this study]
D4381	GCA_005250905.2	Unknown	Unknown	United States	CFF/CFVI [this study]
D5332	GCA_005133705.2	Unknown	Unknown	United States	CFF/CFVI [this study]
D5375	GCA_005250865.2	Unknown	Unknown	United States	CFF/CFVI [this study]
D5675	GCA_005137355.2	Unknown	Unknown	United States	CFF/CFVI [this study]
D7037	GCA_005014375.2	Unknown	Unknown	United States	CFF/CFVI [this study]
INIA-17144	GCA_007723545.1	Ovine	Placenta	Uruguay	CFF/CFVI [this study]
U10	GCF_007109235	Bovine	Prepuce	Uruguay	CFF/CFVI [this study]
97-608	CP008810-CP008812	Bovine	Placenta	Argentina	CFV [10]
98-25	LRES00000000	Bovine	Fetal abomasal content	Argentina	CFV [10]
B6	AJMC00000000	Bovine	Vagina	Australia	CFV [10]
LMG6570	LREW00000000	Bovine	Unknown	Belgium	CFV [10]
66Y	JPQC00000000	Bovine	Prepuce	Canada	CFV [10]
TD	JPPC00000000	Bovine	Prepuce	Canada	CFV [10]
CCUG 33900	LREV00000000	Bovine	Abortion	France	CFV [10]
07BS020	ERS686635	Bovine	Prepuce	Germany	CFV [10]
09CS0030	ERS686637	Bovine	Prepuce	Germany	CFV [10]
11CS0190	ERS686638	Bovine	Prepuce	Germany	CFV [10]
11CS0191	ERS686639	Bovine	Prepuce	Germany	CFV [10]
13CS0183	ERS686640	Bovine	Prepuce	Germany	CFV [10]
14CS0001	ERS686641	Bovine	Prepuce	Germany	CFV [10]
BS 201-02	ERS686632	Bovine	Prepuce	Germany	CFV [10]
BS 38-06	ERS686634	Bovine	Prepuce	Germany	CFV [10]
BS 76-04	ERS686633	Bovine	Fetus	Germany	CFV [10]
C1	ERS739275	Bovine	Prepuce	Spain	CFV [10]
C19	ERS739293	Bovine	Prepuce	Spain	CFV [10]
C2	ERS739276	Bovine	Prepuce	Spain	CFV [10]
C22	ERS739296	Bovine	Prepuce	Spain	CFV [10]
C23	ERS739297	Bovine	Prepuce	Spain	CFV [10]
C24	ERS739298	Bovine	Prepuce	Spain	CFV [10]
C25	ERS739299	Bovine	Prepuce	Spain	CFV [10]
C27	ERS739301	Bovine	Prepuce	Spain	CFV [10]
C30	ERS739304	Bovine	Prepuce	Spain	CFV [10]
C7	ERS739281	Bovine	Prepuce	Spain	CFV [10]
NCTC 10354	CM001228	Bovine	Mucus	United Kingdom	CFV [10]
84-112	HG004426-HG004427	Bovine	Genital secretion	United States	CFV [10]
B10	LRET00000000	Bovine	Unknown	United States	CFV [10]
CFV08A1102-42A	GCA_011600845.2	Bovine	Prepuce	Canada	CFV [This study]
CFV08A948-2A	GCA_011601005.2	Bovine	Prepuce	Canada	CFV [This study]
772	GCA_002973655.1	Human	Ascites	China	n.d.
B1-01	GCA_013184565.1	Human	Blood	China	n.d.
B1-04	GCA_013184585.1	Human	Blood	China	n.d.
wqj1	GCA_010883085.1	Human	Blood	China	n.d.
wqj11	GCA_010883105.1	Human	Blood	China	n.d.
wqj2	GCA_010120605.1	Human	Blood	China	n.d.
wqj3	GCA_010883155.1	Human	Blood	China	n.d.
wqj4	GCA_001699725.1	Human	Blood	China	n.d.
wqj525	GCA_010120605.1	Human	Amniotic fluid/blood	China	n.d.
wqj7	GCA_010883155.1	Human	Blood	China	n.d.
RA36	ERS672213	Reptile	Feces	Italy	n.d.
RA8	ERS672214	Reptile	Feces	Italy	n.d.
11S02557-2	GCA_001699125.1	Reptile	Culture	Netherlands	n.d.
12S00416-3	GCA_001699205.1	Reptile	Unknown	Netherlands	n.d.
12S01208-4	GCA_003994875.1	Reptile	Cloacal swab	Netherlands	n.d.
12S01908-5	GCA_003994885.1	Reptile	Cloacal swab	Netherlands	n.d.
12S02225-3	GCA_001699215.1	Reptile	Unknown	Netherlands	n.d.
12S02263-3	GCA_001699255.1	Reptile	Unknown	Netherlands	n.d.
12S02842-30	GCA_001699265.1	Reptile	Unknown	Netherlands	n.d.
12S02847-1	GCA_001699295.1	Reptile	Unknown	Netherlands	n.d.
12S02855-1	GCA_001699305.1	Reptile	Unknown	Netherlands	n.d.
12S04217-1	GCA_001699335.1	Reptile	Feces	Netherlands	n.d.
12S05168	GCA_001699175.1	Reptile	Unknown	Netherlands	n.d.
13S00388-15	GCA_001699135.1	Reptile	Unknown	Netherlands	n.d.
0006027	ERS739269	Human	Blood	Taiwan	n.d.
0008764	ERS739268	Human	Blood	Taiwan	n.d.
pet-3	GCA_000814265.1	Reptile	Feces	Taiwan	n.d.
CF78-2	GCA_001699365.1	Reptile	Unknown	United Kingdom	n.d.
Sp3	GCA_001484645.1	Reptile	Cell culture	United Kingdom	n.d.
2016D-0237	GCA_005014935.2	Unknown	Unknown	United States	n.d.
2016D-0238	GCA_005255865.2	Unknown	Unknown	United States	n.d.
85-387	GCA_001699345.1	Reptile	Unknown	United States	n.d.
D4335	GCA_001699385.1	Human	Feces	United States	n.d.
D6659	GCA_001699415.1	Human	Pleural fluid	United States	n.d.
D6683	GCA_001699425.1	Human	Hematoma	United States	n.d.
D6690	GCA_001699455.1	Human	Blood	United States	n.d.
D6783	GCA_001699465.1	Human	Feces	United States	n.d.
D6856	GCA_001699485.1	Human	Bile	United States	n.d.
MGYG-HGUT-02374	GCA_902386455.1	Human	Gut	United States	n.d.
03-427	GCA_000495505.1	Human	Unknown	Unknown	n.d.

*C. fetus=Campylobacter fetus,* n.d.=Not determined
